# Association of Cytokine and Toll-Like Receptor Gene Polymorphisms with Severe Malaria in Three Regions of Cameroon

**DOI:** 10.1371/journal.pone.0081071

**Published:** 2013-11-27

**Authors:** Tobias O. Apinjoh, Judith K. Anchang-Kimbi, Clarisse Njua-Yafi, Regina N. Mugri, Andre N. Ngwai, Kirk A. Rockett, Eric Mbunwe, Richard N. Besingi, Taane G. Clark, Dominic P. Kwiatkowski, Eric A. Achidi, in collaboration with The MalariaGEN Consortium

**Affiliations:** 1 Department of Biochemistry and Molecular Biology, University of Buea, Buea, Cameroon; 2 Department of Zoology and Animal Physiology, University of Buea, Buea, Cameroon; 3 Department of Animal Biology and Physiology, University of Yaounde I, Yaounde, Cameroon; 4 Department of Medical Laboratory Sciences, University of Buea, Buea, Cameroon; 5 Wellcome Trust Centre for Human Genetics, University of Oxford, Oxford, United Kingdom; 6 Wellcome Trust Sanger Institute, Hinxton, United Kingdom; 7 Diabetes Research Center, Brussels Free University, Brussels, Belgium; 8 Department of Oral Biology, University of Florida, Gainesville, Florida, United States of America; 9 London School of Hygiene and Tropical Medicine, London, United Kingdom; Wake Forest University Health Sciences, United States of America

## Abstract

*P. falciparum* malaria is one of the most widespread and deadliest infectious diseases in children under five years in endemic areas. The disease has been a strong force for evolutionary selection in the human genome, and uncovering the critical human genetic factors that confer resistance to the disease would provide clues to the molecular basis of protective immunity that would be invaluable for vaccine development. We investigated the effect of single nucleotide polymorphisms (SNPs) on malaria pathology in a case- control study of 1862 individuals from two major ethnic groups in three regions with intense perennial *P. falciparum* transmission in Cameroon. Twenty nine polymorphisms in cytokine and toll-like receptor (TLR) genes as well as the sickle cell trait (HbS) were assayed on the Sequenom iPLEX platform. Our results confirm the known protective effect of HbS against severe malaria and also reveal a protective effect of SNPs in interleukin-10 (IL10) cerebral malaria and hyperpyrexia. Furthermore, *IL17RE* rs708567 GA and *hHbS* rs334 AT individuals were associated with protection from uncomplicated malaria and anaemia respectively in this study. Meanwhile, individuals with the hHbS rs334 TT, *IL10* rs3024500 AA, and *IL17RD* rs6780995 GA genotypes were more susceptible to severe malarial anaemia, cerebral malaria, and hyperpyrexia respectively. Taken together, our results suggest that polymorphisms in some immune response genes may have important implications for the susceptibility to severe malaria in Cameroonians. Moreover using uncomplicated malaria may allow us to identify novel pathways in the early development of the disease.

## Introduction

Malaria affects about one quarter of a billion people annually, with up to two-thirds of a million deaths still occurring per year, particularly in Sub-Saharan African children below five years of age [1]. Why only a small proportion (1–3%) of *P. falciparum* infections progress to severe or fatal episodes [2] while others remain asymptomatic or develop an uncomplicated illness is not yet fully understood. Innate immune recognition of *Plasmodium* and subsequent release of cytokines are known to be important for parasite clearance but may also contribute to disease severity [3]. Furthermore, epidemiological data indicate that about 25% of the risk to *Plasmodium* infection in Africa is determined by human genetic factors [4] and it seems likely that inherent genetic differences in people’s control of immune responses may partly determine the outcome of the disease [5].

Several studies have demonstrated that the imbalance of pro- and anti-inflammatory cytokines is associated with the immuno-pathogenesis of severe malaria anaemia (SMA) and cerebral malaria (CM) [6-9] with Tumor necrosis factor (TNFα) and interleukin-10 (IL10) critical in this role. Therefore, unique functional polymorphisms in the promoter and/or coding region(s) of cytokine genes [8,10] may be critical in the development and clinical course of malaria. Indeed, polymorphisms in genes encoding IL10, IL4 and TNFA [11,12] have been associated with susceptibility to disease. However, the functional role of TNF-promoter polymorphisms that are associated with severe malaria [13-15] still remains open to question [11,15,16] especially as the surrounding MHC class III region has many other interesting immunological genes and complex patterns of linkage disequilibrium [17]. Thus, although TNFα is unquestionably an important mediator of malaria immunity and pathogenesis, it remains possible that the observed genetic associations with *TNFA* polymorphisms arise from functional variation in neighbouring genes [16,18] rather than TNF itself.

Several lines of evidence have linked *IL10* promoter polymorphisms with differential production and expression of IL10 in a number of disease states [19,20]. However, susceptibility to SMA and functional changes in circulating IL10 concentrations has been associated with polymorphic variability in*IL10* promoter haplotypes but not individual loci [8]. Furthermore, an analysis of *IL10* single nucleotide polymorphisms (SNPs) in Gambian children found a common haplotype that was strongly associated with protection against severe malaria by case-control analysis but not by Transmission Disequilibrum Test (TDT) analysis of the same population [21]. The association of *IL10* with severe malaria might be confounded by foetal survival rates or other sources of transmission bias, since genetic variation at the *IL10* locus has been implicated as a determinant of fertility [22]. 

Analysis of SNPs in the *IL22* gene found several weak associations with severe malaria in Gambian children but no clear cut effect [23] while a SNP in *IL1A* (encoding interleukin1α) and another in *IL1B* (encoding interleukin-1β) showed a marginal association with susceptibility to malaria [24]. The role of SNPs in cytokine genes in the development of severe malaria severity thus remains unclear. 

The initial trigger for the production or overproduction of cytokines is thought to depend on the type of interaction between host-cells’ pattern-recognition receptors and the parasite [5]. The family of Toll-like receptors (TLR) which recognize *Plasmodium* motifs and induce the innate immune system to initiate an inflammatory response [25-27] may be critical in severe malaria pathogenesis. *P. falciparum glycosylphosphatidylinositol* has been reported to induce signaling via both TLR4 [25] and haemozoin-induced immune activation was shown to involve TLR9 [28,29]. Activation of TLR9 expressed on dendritic cells has also been proposed as a mechanism used by malaria parasites to trigger regulatory T-cells to evade the immune system [30]. SNPs in *TLR2*, -4, and -9, have been associated with susceptibility to infectious and inflammatory diseases (reviewed in 31). Two frequently co-segregating *TLR4* polymorphisms were observed to reduce reactivity to inhaled lipopolysaccharide [32], although findings are partly conflicting [33]. *TLR4* variants have been shown to increase the risk to septic shock [34], Gram-negative infections [35-37] as well as severe malaria in children [26]. Although *TLR9* promoter polymorphisms have been associated with asthma [38], Crohn’s disease [39], clinical manifestation of malaria during pregnancy [27] and high parasitaemia in a cohort of patients with uncomplicated malaria [40], no clear associations have been observed with severe malaria [26,41]. There is need therefore for additional studies to elucidate the role of TLRs in severe malaria pathogenesis.

Case–control studies have been vital in detecting several genes associated with severe malaria [15,42-44]. However, some reports have been contradictory, partly due, to the analysis of small sample numbers, and hence limited statistical power. Furthermore, differences in transmission intensities or other epidemiologic characteristics at the different sites and ethnicities may affect the detection of modest effects of susceptibility or resistance genes. We therefore examined known and frequent cytokine and *TLR* SNPs in a case-control study among 971 children with malaria and 891 unmatched apparently healthy control school children and blood bank donors. 

## Methods

### Ethics Statement

Ethical clearance for the study was obtained from the University of Buea Institutional Review Board and the South West Regional Delegation of Public Health. Authorization to conduct the surveys in designated primary schools was obtained from the Regional Delegation of Basic Education or the Catholic Education Secretariat of the South West Region. Individuals who fulfilled the specific inclusion criteria and volunteered to participate after adequate sensitisation on the project objectives, methods and possible benefits/risks were enrolled into the study. A health facility or school was only investigated with the approval of its Director or Headteacher and study participants were only enrolled if they or their caregivers/guardians gave written informed consent/assent. 

### Study participants

Patient samples were collected as part of epidemiological studies of severe malaria in children from the Centre, Littoral and South-West regions of Cameroon, conducted between 2003/5 and 2007/8 [9,45,46]. The sample of 971 unrelated sick children included severe and uncomplicated malaria cases, mainly from the Bantu and Semi-Bantu ethnic groups, recruited from the paediatric wards of nine hospitals and health centres (Table 1). In line with WHO guidelines [47], severe malaria was defined as the presence of asexual *Plasmodium* parasitaemia and at least one of the following conditions: Cerebral Malaria (CM) [impaired consciousness or unrousable coma (Blantyre coma score ≤ 2) and no record of recent severe head trauma, neurological disease or any other cause of coma]; Severe Malarial Anaemia (SMA) [haemoglobin < 5g/dL or haematocrit < 15%, no cases of severe bleeding or observed convulsions]; hyperpyrexia (axillary temperature ≥ 40°C); hyperparasitaemia (>250,000 parasites/µL); convulsions before/during admission; respiratory distress (presence of alar flaring, intercostals or subcostal chest recession, use of accessory muscles of respiration, or abnormally deep respiration) and hypoglycaemia (blood glucose <2.2mmol/L); Anaemia (haemoglobin < 11 g/dL or haematocrit < 33%). Participants with co-existing severe or chronic medical conditions (e.g. bacterial pneumonia, kwashiorkor) unrelated to a severe malarial infection were excluded. Uncomplicated Malaria (UM) was defined as a clinical illness characterized by an axillary temperature ≥ 37.5°C associated with a *Plasmodium* positive blood film, haemoglobin ≥ 8g/dL and full consciousness, in the absence of clinical signs and symptoms of severe malaria and/or evidence of vital organ dysfunction.

**Table 1:** Basic demographic and clinical characteristics of the study population.

**Variable**	**Cases of malaria**	**Control**
	**n (%)**	**n (%)**
**All subjects**	971 (49.4)	891 (45.4)
**Age^§^** (Mean ± SD)		
Children	55.40 ± 37.89	87.69 ± 22.89
Adults	-	346.06 ± 91.9
**Gender**		
Female	453 (46.7)	294 (33.1)
Male	516 (53.3)	594 (66.9)
**Ethnicity**		
Bantu	385 (46.3)	392 (47.5)
Foulbe	31 (3.7)	17 (2.1)
Semi-Bantu	416 (50.0)	417 (50.7)
**Location**		
Centre	164 (16.9)	404 (45.3)
Littoral	292 (30.1)	-
South West	515 (53.0)	487 (54.7)
**Phenotype**		
Any severe malaria	484 (49.9)	
SMA	248 (21.8)	
CM	51 (5.4)	
CM + SMA	11 (1.2)	
Hyperpyrexia	116 (15)	
Hyperparasitaemia	58 (6.5)	
UM	252 (22.1)	

^§^ in months, SMA = severe malarial anaemia, CM = cerebral malaria, UM = uncomplicated malaria

Controls (n=891) consisted of apparently healthy children (aged 1-14 years, afebrile and free from any obvious illness) and adults (aged 17-52 years, asymptomatic, from the community) also belonging primarily to the Bantu and Semi-Bantu ethnic groups (Table 1). Children were recruited during malaria cross-sectional surveys from primary schools located in the South-West region (Buea Metropolis) between 2004-2005 and 2007-2008. Children with parasitaemia and a temperature of 37.5°C or above were not recruited as controls. Adults were identified from a blood bank in the centre region (Mother and Child Hospital - Yaounde) between July and August 2007. These controls were thought to approximate a random sample of the population thus, reflecting the true polymorphisms’ allele frequencies. Data on the Foulbe ethnic group who constitute a significant proportion of the ethno-demographics of Cameroon were not included in the final analysis of data because of their small sample size in the study population. 

### Malaria parasitaemia determination

Thick and thin blood smears were prepared following standard procedures and stained with 5% Giemsa (Sigma, St. Louis, USA). The malaria parasitaemia status and density were determined under oil immersion with the 100x objective, plus a 10x eyepiece of a binocular Olympus microscope (Olympus Optical Co., Ltd, Japan) while the *Plasmodium* species was identified on the thin blood smear. A smear was only considered negative if no malaria parasites were seen in 50 high-power fields. With each positive smear, the level of parasitaemia was estimated by counting the parasites against at least 200 leucocytes and using the corresponding leucocyte count to calculate the number of parasites/µl blood [48].

### Cytokine measurement

Plasma levels of IL10 and TNFα were measured in cases and controls by Enzyme-Linked Immunosorbent Assay (ELISA), according to instructions from the manufacturer (Quantikine R&D systems). Samples were obtained at the time of admission (CM/SMA), outpatient treatment (UM), or enrollment (Controls). The results were expressed in pg/ml by reference to standard curves prepared in each plate with recombinant cytokines. In all the ELISAs, standards/samples were run in duplicates, and tested 10 non-immune Swedish & British sera were used as negative controls to check that the response was specific to malaria infection.

### Selection of SNPs and genotyping

Genomic DNA was extracted from whole blood or packed cells using the Promega Wizard (Promega Corporation, Madison, USA) or Nucleon™ BACC Genomic DNA Extraction (Gen-Probe Life Sciences, Manchester, UK) kits and quantified by the picogreen assay. The DNA samples were whole-genome amplified by Primer Extension Pre-amplification [49] before genotyping on the Sequenom IPLEX genotyping platform (Sequenom Inc., San Diego, USA) [50]. Polymorphism sequence information was downloaded from Ensembl (http://www.ensembl.org) and reformatted for the assay design process (www.sequenom.com). Multiplex design for the iPLEX methodology was then undertaken using the MassARRAY^®^ Assay Design v3.1 Software and the resulting multiplexes tested using a panel of CEPH and YRI HapMap DNAs. Thirty malaria candidate polymorphisms in cytokine (IL1, IL10, IL13, IL17, IL20, IL22, IRF1, LTA & TNFA) and Toll-like receptor (TLR1, TLR4, TLR6 & TR9) genes were genotyped in 971 cases and 891 controls. The SNPs were compiled on the basis of a review of reports of associations with severe malaria or association with other infectious diseases. The haemoglobin variant S (HbS) polymorphism, known to be strongly associated with severe malaria [18,42] was also genotyped. Genotype calling was performed using the Sequenom Spectro-typer software v4.2 (an automated algorithm), followed by a careful visual inspection of the genotype cluster plots to assess quality. All the assays passed the quality filters (call rate >90%), with no evidence of genotypic deviation from Hardy-Weinberg equilibrium (HWE) in the controls (P<0.001), indicative of genotyping error [51].

### Data Analyses

Statistical analyses were performed using IBM SPSS Statistics 17.0 (IBM Corporation, NY, USA) and the R software package (http://www.r-project.org). Genotypic deviations from HWE were assessed using the Pearson’s Chi-square statistical test. Case-control association analysis of SNP alleles and genotypes was undertaken by logistic regression with self-reported ethnicity, age, and the HbS polymorphism included as covariates. Adjustment for self-reported ethnicity has been found to be a robust approach to controlling the potentially confounding effects of population structure [42]. Performing multiple statistical tests can lead to an increased chance of false positives. Thus, for the genetic results, we performed a simulation by permuting the phenotypes 10000 times, and found a significance threshold of P ≤ 0.01 to be equivalent to a nominal false positive rate of 5%.

## Results

The basic demographic and clinical characteristics of the study population are shown (Table 1). One SNP was removed from the analysis because it was monomorphic (rs5743611), leaving 29 SNPs that could be analyzed for their association with severe malaria (Table 2). All the assays passed the quality filters (call rate <90%), with no evidence of genotypic deviation from HWE in the controls.

**Table 2:** Minor allele frequencies and Test of Hardy-Weinberg Equilibrium in selected candidate SNPs.

**Gene**	**Alternate SNP Name**	**SNPs**	**Location**	**Minor Allele Frequency**			
				Maj/Min	**Cases (n = 871)**	**Controls (n = 891)**	**HWE^†^ (P value)**
HBB	HbS	rs334	Genic	A/T	0.054	0.087	0.07
IL10		rs3024500	P	**A/G**	0.401	0.412	0.94
IL10	IL-10 -1082	rs1800896	UTR	C/T	0.329	0.307	0.29
IL10	IL-10 -3533	rs1800890	UTR	A/T	0.230	0.251	0.14
IL1A	IL-1A G4845T	rs17561	Genic	**G/T**	0.128	0.147	0.02
IL1B	IL-1B A2	rs1143634	Genic	**C/T**	0.123	0.138	0.16
IL17RE		rs708567	Genic	A/G	0.485	0.495	0.64
TLR9		rs187084	P	C/T	0.256	0.296	0.51
IL17RD		rs6780995	Genic	A/G	0.429	0.430	0.36
TLR1		rs4833095	Genic	C/T	0.108	0.107	0.86
TLR6		rs5743810	Genic	C/T	0.001	0.001	1.00
TLR6		rs5743809	Genic	C/T	0.041	0.055	0.51
IRF1		rs2706384	P	A/C	0.363	0.389	0.50
IL13	IL-13 46457	rs20541	Genic	C/T	0.188	0.157	0.05
IL4	IL-4 -589	rs2243250	I	C/T	0.210	0.199	0.001
LTA	LTA+80	rs2239704	Genic	G/T	0.237	0.265	0.65
LTA	LTA NCOI	rs909253	Genic	C/T	0.431	0.444	0.52
TNF	TNF -1031	rs1799964	P	C/T	0.128	0.115	0.17
TNF	TNF -376	rs1800750	P	A/G	0.055	0.041	1.00
TNF	TNF -308	rs1800629	P	A/G	0.084	0.078	0.36
TNF	TNF -238	rs361525	P	A/G	0.046	0.046	0.72
TNF	TNF +851	rs3093662	Genic	A/G	0.081	0.083	0.03
IL20RA		rs1555498	Genic	C/T	0.376	0.371	0.65
TLR4		rs4986790	Genic	A/G	0.081	0.061	0.12
TLR4		rs4986791	Genic	C/T	0.005	0.002	1.00
IL22	IL-22 +4583	rs2227507	Genic	C/T	0.036	0.031	0.62
IL22	IL-22 +2611	rs1012356	Genic	A/T	0.465	0.496	0.67
IL22	IL-22 +708	rs2227491	Genic	C/T	0.395	0.382	0.31
IL22	IL-22 -485	rs2227485	P	A/G	0.451	0.449	0.84
IL22	IL-22 -1394	rs2227478	P	A/G	0.382	0.378	0.54

*UTR* 3’untranslated region, *P* promoter, Maj/ Min=Major/Minor allele**. ^†^**One degree of freedom χ^2^ test of HWE applied to the 891 controls

Table 3 shows the minimum p-values from allelic/genotypic tests applied to the autosomal SNPs, and confirms that the sickle cell (HbS) polymorphism (rs334) was significantly associated with protection from malaria infection in heterozygotes [AT vs AA/TT, odds ratio (OR) = 0.34 , 95%CI 0.20-0.58, p=3.08 x 10^-5^ ].Interestingly, this association with hHbS was only found in the Semi-Bantu and not Bantu (Table S2 in File S1) A mutation in 17RE gene was associated strong protection OR (95%CI): 0.67 (0.49-0.93) and 0.58 (0.38 -0.88)from malaria infection in general (Table 3) and UM (Table 4) respectively. Furthermore, carriage of the sickle cell trait was associated with protection from anaemia [odds ratio (OR) = 0.50, 95%CI 0.31-0.80, p=0.004] while a polymorphism in the *IL10* gene was associated with protection, albeit weak, from severe malaria (Table 4).The presence of the rs1800896 SNP in the gene encoding IL10 was associated with decreased risk of cerebral malaria (TT vs CT vs CC, OR=0.41, 95%CI 0.19-0.88, p=0.017) and while rs1800896 CT/CC individuals were more protected from hyperpyrexia (OR=0.60, 95%CI 0.40-0.90, p=0.014) (Table 4). 

**Table 3:** Results of allelic and genotype associations between selected SNPs and malaria.

**Gene**	**SNPs**	**Allele-based tests**						**Genotype-based tests**							
		**Alleles**	**OR**	**95% CI**		**P value**	**Model**	**Genotypes**	**OR**	**95% CI**		**P value (Unadjusted)**	**OR**	**95% CI**	**P value^‡^ (Adjusted**)
HBB	rs334	T vs A	0.60	0.46	0.79	**<0.001**	Heterozygous	AT vs AA/TT	0.30	0.21	0.43	**1.55 x 10^-12^**	0.34	0.20-0.58	**3.08 x 10^-5^**
IL10	rs3024500	G vs A	0.96	0.84	1.10	0.55	Dominant	AG/GG vs AA	1.08	0.82	1.41	0.59	1.19	0.78-1.81	0.41
IL10	rs1800896	T vs C	1.11	0.96	1.29	0.15	Additive	TT vs CT vs CC	1.12	0.96	1.30	0.14	0.98	0.71-1.35	0.91
IL10	rs1800890	T vs A	0.89	0.76	1.04	0.16	Additive	TT vs AT vs AA	0.90	0.76	1.05	0.18	0.83	0.64-1.07	0.15
IL1A	rs17561	T vs G	0.86	0.71	1.04	0.12	Recessive	GG vs GT/TT	0.48	0.24	0.97	**0.035**	0.49	0.18-1.34	0.16
IL1B	rs1143634	T vs C	0.87	0.71	1.06	0.17	Recessive	CC vs CT/TT	0.61	0.30	1.25	0.17	0.60	0.20-1.83	0.37
IL17RE	rs708567	G vs A	0.92	0.81	1.05	0.23	Recessive	AA vs GA/GG	1.20	0.95	1.50	0.12	0.67	0.49-0.93	**0.01**
TLR9	rs187084	C vs T	0.82	0.71	0.96	**0.011**	Additive	CC vs CT vs TT	1.23	1.05	1.44	**0.010**	1.09	0.61-1.97	0.77
IL17RD	rs6780995	G vs A	1.00	0.88	1.15	0.96	Recessive	AA vs GA/GG	1.08	0.88	1.34	0.45	1.38	0.92-2.06	0.12
TLR1	rs4833095	T vs C	1.01	0.81	1.25	0.94	Heterozygous	CT vs TT/CC	1.04	0.80	1.33	0.80	0.87	0.59-1.30	0.51
TLR6	rs5743810	T vs C	1.92	0.17	21.23	0.59	Additive	TT vs CT vs CC	2.07	0.19	22.95	0.54	0.78	0.06-10.37	0.85
TLR6	rs5743809	T vs C	0.75	0.54	1.03	0.07	Additive	TT vs CT vs CC	0.72	0.51	1.01	0.05	0.72	0.41-1.28	0.27
IRF1	rs2706384	C vs A	0.90	0.78	1.03	0.13	Additive	CC vs AC vs AA	0.86	0.74	1.00	0.05	0.77	0.61-0.98	**0.03**
IL13	rs20541	T vs C	1.24	1.03	1.48	**0.020**	Dominant	CT/CC vs TT	1.38	1.11	1.72	**0.003**	1.12	0.79-1.59	0.52
IL4	rs2243250	T vs C	1.07	0.90	1.26	0.45	Heterozygous	CT Vs TT/CC	1.22	0.98	1.52	0.08	0.82	0.62-1.08	0.16
LTA	rs2239704	T vs G	0.86	0.74	1.01	0.06	Dominant	GT/GG vs TT	0.85	0.70	1.04	0.11	1.12	0.80-1.56	0.50
LTA	rs909253	T vs C	0.95	0.83	1.09	0.45	Recessive	CC vs CT/TT	1.11	0.89	1.37	0.35	0.93	0.66-1.31	0.67
TNF	rs1799964	T vs C	1.12	0.91	1.38	0.27	Dominant	CT/CC vs TT	1.16	0.92	1.48	0.20	1.37	0.92-2.04	0.12
TNF	rs1800750	G vs A	1.36	0.99	1.87	0.06	Additive	GG vs GA vs AA	1.51	1.08	2.11	**0.014**	1.47	0.85-2.55	0.17
TNF	rs1800629	G vs A	1.09	0.85	1.39	0.50	Dominant	GA/AA vs GG	1.08	0.82	1.42	0.59	0.88	0.57-1.37	0.58
TNF	rs361525	G vs A	1.01	0.73	1.39	0.96	Heterozygous	AG vs AA/GG	1.10	0.78	1.55	0.59	1.17	0.69-2.00	0.56
TNF	rs3093662	G vs A	0.98	0.77	1.25	0.87	Recessive	AA vs AG/GG	5.44	0.65	45.70	0.06	0.88	0.57-1.35	0.56
IL20RA	rs1555498	T vs C	1.02	0.89	1.18	0.73	Heterozygous	CT vs TT/CC	1.12	0.91	1.36	0.28	1.16	0.84-1.61	0.37
TLR4	rs4986790	G vs A	1.34	1.03	1.75	**0.028**	Dominant	AG/AA vs GG	1.37	1.02	1.84	**0.035**	1.10	0.13-9.17	0.92
TLR4	rs4986791	T vs C	2.47	0.65	9.31	0.18	Additive	TT vs CT vs CC	2.39	0.62	9.29	0.19	2.30	0.19-27.62	0.49
IL22	rs2227507	T vs C	1.18	0.82	1.72	0.38	Additive	TT vs CT vs CC	1.23	0.82	1.83	0.31	1.24	0.62-2.47	0.54
IL22	rs1012356	T vs A	0.86	0.75	0.98	**0.025**	Dominant	TA/AA vs TT	0.80	0.64	1.00	0.05	0.92	0.73-1.14	0.43
IL22	rs2227491	T vs C	1.05	0.92	1.21	0.46	Recessive	CC vs CT/TT	0.94	0.77	1.15	0.55	0.86	0.57-1.30	0.46
IL22	rs2227485	G vs A	1.01	0.88	1.16	0.86	Heterozygous	GA vs GG/AA	0.91	0.74	1.11	0.33	0.96	0.68-1.35	0.81
IL22	rs2227478	G vs A	1.02	0.88	1.17	0.83	Dominant	GA/AA vs GG	1.07	0.81	1.42	0.65	0.91	0.65-1.26	0.55

*OR* odds ratios, *CI* confidence interval, Maj=Major allele; Min=Minor allele**. ^‡^**We performed additive, dominant, recessive and heterozygous advantage genotypic tests, adjusted for age, sex, ethnicity and HbS, but only the most statistically significant result is presented

**Table 4:** Results of genotype associations between selected SNP and syndromes of malaria.

**Phenotype**	**Gene**	**SNPs**	**Model**	**Genotypes**	**OR**	**95% CI**		**P value^‡^**
Anemia	hHbS	rs334	Heterozygous	AT vs AA/TT	0.50	0.31	0.80	**0.004**
	IL4	rs2243250	Additive	TT vs CT vs CC	0.77	0.59	1.00	0.047
CM	IL10	rs1800896	Additive	TT vs CT vs CC	0.41	0.19	0.88	0.017
	IL10	rs3024500	Recessive	AA vs GA/GG	4.20	1.54	11.45	**0.003**
Hyperparasite	IRF1	rs2706384	Heterozygous	AC vs AA/CC	1.93	1.06	3.52	0.028
	TLR1	rs4833095	Dominant	CT/CC vs TT	2.10	1.12	3.94	0.027
Hyperpyrexia	IL10	rs1800896	Dominant	CT/CC vs TT	0.60	0.40	0.90	0.014
	TLR9	rs187084	Additive	CC vs CT vs TT	1.46	1.02	2.08	0.035
	IL17RD	rs6780995	Heterozygous	GA vs GG/AA	1.94	1.27	2.96	**0.002**
SMA	hHbS	rs334	Recessive	TT vs AT/AA	218.52	30.28	1577.2	**7.11 x 10^-8^**
UM	IL17RE	rs708567	Heterozygous	GA vs GG/AA	0.58	0.38	0.88	**0.011**
	IL17RD	rs6780995	Dominant	GA/AA vs GG	1.94	1.04	3.63	0.031

‡ We performed additive, dominant, recessive and heterozygous advantage genotypic tests, adjusted for age, sex, ethnicity and HbS, but only the most statistically significant result is presented. UM = Uncomplicated malaria; Hyperparasite: Hyperparasitaemia (>250000 parasites/ul).

Sickle celled individuals were more likely to develop malaria infection [rs334 TT vs AT/AA, OR=41.33, 95%CI 6.48-263.63, p=8.56 x 10^-7^] and SMA [OR=218.52, 95%CI 30.28-1577.2, p=7.11 x 10^-8^] (Table 4). The CT/CC genotype for the rs20541 SNP in *IL13* locus (OR=1.38, 95%CI 1.11-1.76, p=0.003) and the TLR9 rs187084 SNP (additive T, OR=1.23, 95%CI 1.05-1.44, p=0.010) were associated with susceptibility to malaria (Table 3) following univariate analysis. Moreover, carriage of the rs3024500 SNP in the gene encoding IL10 was associated with an increased risk of CM (additive T, OR=4.20, 95%CI 1.54-11.45, p=0.003) while mutations in the genes encodingIL-17RD (rs6780995) were strongly associated with increased risk of hyperpyrexia (GA vs GG/AA, OR=1.94, 95%CI 1.27-2.96, p=0.002). In addition, a number of genotype associations, albeit weak, were also observed with UM and hyperparasitaemia (Table 4). Individuals with GA/AA genotype in the rs6780995 SNP of IL17RD were protected from UM (OR=1.94, 95%CI 1.04-3.63, p=0.031) while IRF1 rs2706384 heterozygotes (OR=1.93, 95%CI 1.06-3.52, p=0.028) and CT/CC genotypes of TLR1 rs4833095 (OR=2.10, 95%CI 1.12-3.94, p=0.027) were more refractory to hyperparasitaemia.

**Figure 1:**
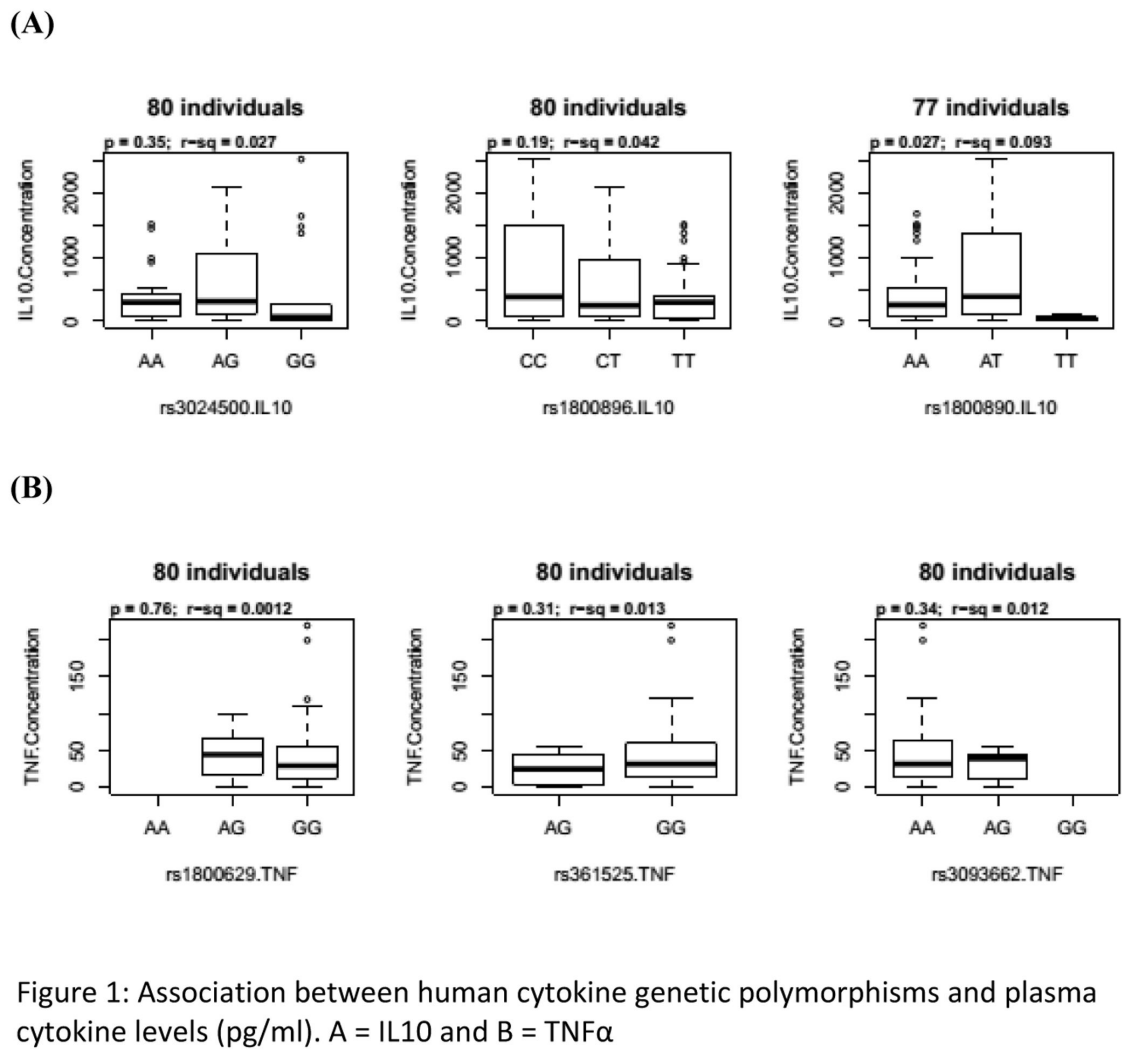
Association between human cytokine genetic polymorphisms and plasma cytokine levels. Plasma cytokine levels measured by ELISA were compared across human IL10 and *TNF* SNP genotypes of study participants. Of the three polymorphisms assayed for each cytokine, only the AA and TT genotypes of IL10 rs1800890 had lower plasma IL10 levels compared to their AT counterparts.

Plasma IL10 levels were strongly correlated with the heterozygous AT genotype of *IL10* rs1800890 SNP (p=0.027, Figure 1A). The AA and TT genotypes of IL10 rs1800890 had lower plasma IL10 levels compared to their AT counterparts. Nevertheless, no other *IL10* (Figure 1A) or *TNF* (Figure 1B) SNP genotypes were significantly associated with IL10 or TNFα plasma levels, respectively.

## Discussion

There is compelling evidence that the human genome has evolved under selective pressure exerted by microbial pathogens. Perhaps one of the best characterized examples of this natural selection process is the historical exposure to *falciparum* malaria and its association with the β-globin SNP underlying the haemoglobin S sickle-cell trait. The frequency of this mutant allele can reach a frequency of 25% in some populations in Sub-Saharan Africa where it has been observed to mediate a strong protective effect against severe malaria [18]. Data reported here provide additional understanding on how innate immunity may contribute to malaria susceptibility. Furthermore, data will help define the basis of individual and population variation in susceptibility to malaria pathogenesis that may eventually facilitate efforts to develop vaccines and treatments to fight this infectious disease.

This study focused on cytokine and Toll-like receptor genes given that a crucial balance between the induction of an inflammatory response against infection and inflammopathology appears to be a key to severe disease progression [5]. Evidence for this includes *in vitro* experiments in which engagement of TLRs by putative malaria toxins and activation of downstream signalling pathways lead to production of pro-inflammatory cytokines [25,29], and from case-control studies in which the frequencies of selected polymorphisms among individuals with severe malaria illness are compared with those of ethnically and age-matched residents with asymptomatic malaria [18,26]. Our data demonstrate the first evidence that polymorphisms in *IL17* may be associated with uncomplicated malaria in the Cameroonian population while SNPs in, *IL10*, *IL17RD*, *IRF1*, *TLR1*and *TLR9* may be linked with severe malaria in general. 

One important difference between our study and others is that the number of CM cases was quite low. Consequently, mutations associated with susceptibility/resistance to this phenotype were unlikely to be found. On the other hand, geographic differences in parasitological or ethnic parameters could influence the level of resistance/susceptibility observed. 

The activation of dendritic cells by malaria schizonts has been demonstrated to involve TLR9 [29]. Our observed susceptibility association with *TLR9* SNPs parallel previous reports of increased risk of low birth weight in term infants of malaria infected pregnant women carrying the *TLR9* T-1486C polymorphisms [27]. However, a recent study reported only a weak association of *TLR9* polymorphism with severe malaria in Malawi and The Gambia, which was not replicated in family based cohort analyses [41]. Furthermore, allelic expression imbalances have been observed [41], indicating that different individuals might differ in the expression of *TLR9*, which can lead to different responses to the stimulus induced by malaria motifs. 

Although common *TLR4* mutations have previously been shown to increase the risk of severe malaria in African children [26], we observe only a marginal susceptibility effect of *TLR4* rs4986790 to severe malaria in this study. This suggests that TLR4 contributes to parasite recognition and host responses *in vivo* as well as mediate innate immune responses to *P. falciparum* through the recognition of GPI [25]. In analogy to bacterial infections, *TLR4* polymorphisms may reduce responsiveness to *P. falciparum* and to GPI in particular [26] and thus cause severe malaria due to both inadequate innate responses at disease onset and insufficient stimulation of specific immunity during preceding infections. As with other genetic host factors [10], the role of *TLR* polymorphisms may vary with malaria endemicity, as does disease manifestation. The observed impact of *TLR* variants on malaria among children from a highly endemic area thus needs to be verified in settings of differing malaria transmission and clinical manifestation pattern.

The fact that *IL13* resides in the human chromosome 5q31–33 region and has been shown to influence the clinical outcome of malaria [52] as well as immunoglobulin E (IgE) levels [53] raises the possibility of association of its variants with malaria severity. Previous studies have shown that *IL13*-1055C4T, a promoter polymorphism affects expression levels and is associated with allergic asthma [54] and chronic obstructive pulmonary disease [55]. The marginal susceptibility effect of the C allele and increased risk of the heterozygous genotype observed in this study contradicts previous reports of a protection effect of *IL13*_1055T allele from severe malaria in Thai malaria patients [56]. More analysis in large cohorts shall be required to confirm the role played by this SNP in malaria pathogenesis. It is possible that an apparent association with malaria can arise from linkage disequilibrium between the typed SNP and a primarily associated polymorphism. Constructing a detailed map of polymorphisms around the IL-13 gene and describing a profile of linkage disequilibrium among them will be helpful for further association and functional studies of *IL13* polymorphism with severe malaria.

Previous studies have linked *IL10* promoter polymorphisms with differential production and expression of IL10 in a number of disease states [19,20]. Variation in cytokine promoter sequences, such as the IL-10 promoter, likely alter specific transcription factor recognition sites and consequently affect transcriptional activation and cytokine production [8]. Nevertheless, only rs1800890 associated with IL10 production (Figure 1), although a number of SNPs have been associated with differences in IL10 production [19,20]. This suggests that rs1800890 may act to upregulate IL10 transcription, with the heterozygotes providing some selective advantage since the raised IL10 levels will down-regulate proinflammatory cytokines such as TNFα and protect against severe malaria [9]. Turner et al. demonstrated a difference in IL-10 secretion in association with the presence or absence of of the IL-10−1082*A allele in the human IL-10 promoter, after stimulation of peripheral blood mononuclear cells [57]. This is in accordance with our observation of higher IL-10 plasma levels in individuals with the IL-10 rs1800890 AT genotype compared to those with the homozygotes. Nevertheless, no difference in the plasma IL-10 levels with other IL10 SNPs typed in this study. It should be noted that individual differences in the levels of the IL-10 measured at a specific moment may not only result from host genetic factors predisposing to high or low production, but also for a great part from the physiological condition at that time, as well as from global immunity. However, rs3024500 and rs1800896 were associated with altered risk of CM in this study, contradicting previous observations [21]... Therefore, screening of further case–control groups would be useful to explore whether IL10 variants are involved in malaria susceptibility in other Sub-Saharan African populations. In addition, an appropriately designed family-based study may provide useful linkage information, have more power to detect associations and allow haplotypes to be constructed with more confidence. The possibility also exists that the functional effect in the case of malaria depends on a specific combination of SNPs present. 

IL22 is a member of the IL10 family of cytokines [23] produced mainly by activated Th1 cells. Although it acts primarily to increase the innate immunity of tissues [58], it may play a different role in malaria. Our observed associations with disease severity agree with earlier studies in The Gambia that reported an association of the IL22+708T allele with protection against severe anaemia [23]. In order to detect/confirm such small but important effects when testing many polymorphisms, much larger group sizes will be needed.

Since the transcription factor IRF1 is a critical effector molecule in IFN-gamma signalling, variants may affect human susceptibility to malaria. This is supported by reports of an association between an IRF1 polymorphism and human immunodeficiency virus (HIV) - infection in Kenya [59], with protective genotypes showing reduced gene expression, both before and after IFNγ stimulation. Nevertheless, we detected a susceptibility effect of an *IRF1* polymorphism to hyperparasitaemia, consistent with previous findings of an association of *IRF1* polymorphisms with the control of *P. falciparum* infection (parasite prevalence and density), both in healthy adult and in children with uncomplicated and severe malaria in Burkina Faso [60]. However, no significant associations were observed between the SNPs and severe malaria in The Gambia, Kenya and Malawi [61] as was the case in this study. It might be possible that the association of IRF1 polymorphisms with the ability to control parasitaemia and the lack of association with disease severity actually reflect differences in the molecular mechanisms underlying protective/pathological immune responses at different stages of infection [61]. Furthermore, the effects of *IRF1* polymorphisms on severe malaria may be too small to be detected with this sample size, or may affect only a sub-phenotype of severe malaria.

Signalling pathways in murine malaria are thought to include *IL17*, with Toll-like receptor modulation of murine CM thought to depend on the genetic background of the host [62]. The down-regulation of IL-17RD, an inhibitor of receptor tyrosine kinase signalling has been shown to be common to a variety of human carcinomas [63]. However, no evidence exists yet for raised levels of IL17 in malaria in spite of huge efforts undertaken on CM patients in Ghana [64] and India [65]. Nevertheless, we present the first report of an association between rs6780995 and hyperpyrexia. Further work is needed to consolidate these findings.

## Conclusions

Our genetic-association studies confirm and extend the strong protective effect of the sickle cell trait against malaria as well as show that sticklers are more susceptible to severe malarial anaemia. Our data demonstrate the first evidence that polymorphisms in*IL17* may be associated with uncomplicated malaria in the Cameroonian population while SNPs in, *IL10 IL17RD*, *IRF1, TLR1* and *TLR9* may be linked with severe malaria. These suggest that polymorphisms in human genes may have important implication for outcome to paediatric malaria in Cameroon, possibly by altering the transcription of cytokine genes. Moreover using uncomplicated malaria may allow us to identify novel pathways in the early development of disease. However, it is possible that an apparent association with malaria can arise from linkage disequilibrium between the typed SNP and a primarily associated polymorphism. Constructing a detailed map of polymorphisms around these candidate genes and describing a profile of linkage disequilibrium among them will be helpful to identify the causal variant(s). In addition, an appropriately designed family-based study may provide useful linkage information, have more power to detect association and allow haplotypes to be constructed with more confidence.

## Supporting Information

File S1 **Material S1, Membership of The Malaria Genomic Epidemiology Network (MalariaGEN).** Table S1, Minor Allele Frequencies and Test of Hardy-Weinberg Equilibrium in selected candidate SNPs across ethnic groups. Table S2, Results of genotype associations between selected SNP and syndromes of malaria in the two major ethnic groups.
(DOCX)
