# Supplementary material for: Association of Cytokine and Toll-Like Receptor Gene Polymorphisms with Severe Malaria in Three Regions of Cameroon
Source: PLoS One. 2013 Nov 27;8(11):e81071. doi: 10.1371/journal.pone.0081071 (PMC3842328; doi:10.1371/journal.pone.0081071)
Supplement: File S1 — Material S1, Membership of The Malaria Genomic Epidemiology Network (MalariaGEN). Table S1, Minor Allele Frequencies and Test of Hardy-Weinberg Equilibrium in selected candidate SNPs across ethnic groups. Table S2, Results of genotype associations between selected SNP and syndromes of malaria in the two major ethnic groups. (DOCX) [file pone.0081071.s001.docx]

**SUPPLIMENTARY MATERIALS**

Material S1. Membership of The Malaria Genomic Epidemiology Network (MalariaGEN**)**

**Lead Investigators:** Eric Akum Achidi^1^, Tsiri Agbenyega2, Stephen Allen^3,4^, Olukemi Amodu^5^, Kalifa Bojang^6^, David Conway^6^, Patrick Corran^7^, Panos Deloukas^8^, Abdoulaye Djimde^9^, Amagana Dolo^9^, Ogobara Doumbo^9^, Chris Drakeley^10,11^, Patrick Duffy^12,13^, Sarah Dunstan^14^, Jennifer Evans2,15, Jeremy Farrar14, Deepika Fernando^16^, Tran Tinh Hien^14^, Rolf Horstmann^15^, Muntaser Ibrahim^17^, Nadira Karunaweera^16^, Gilbert Kokwaro^18^, Kojo Koram^19^, Dominic Kwiatkowski^8,20^, Martha Lemnge^21^, Julie Makani^22^, Kevin Marsh^18^, Pascal Michon^3^, David Modiano^23^, Malcolm E. Molyneux^24^, Ivo Mueller^3^, Theonest Mutabingwa^12^, Michael Parker^25^, Norbert Peshu^18^, Chris Plowe^26,27^, Odile Puijalon^28^, Jiannis Ragoussis^20^, John Reeder^3^, Hugh Reyburn^10,11^, Eleanor Riley^10^, Jane Rogers^8^, Anavaj Sakuntabhai^28^, Pratap Singhasivanon^29^, Sodiomon Sirima^30^, Giorgio Sirugo^6^, Adama Tall^31^, Terrie Taylor^26,32^, Mahamadou Thera^9^, Marita Troye-Blomberg^33^, Tom Williams^18^ & Michael Wilson^19^

**Data Fellows:** Lucas Amenga-Etego^19,34^, Tobias O. Apinjoh^1^, Edith Bougouma^30^, Rajika Dewasurendra^16^, Mahamadou Diakite^9^, Anthony Enimil^2^, Ayman Hussein^17^, Deus Ishengoma^21^, Muminatou Jallow^6^, Enmoore Lin^3^, Alioune Ly^31^, Valentina D. Mangano^20,23^, Alphaxard Manjurano^10,11^, Laurens Manning^3^, Carolyne M. Ndila^18^, Vysaul Nyirongo^24^, Tom Oluoch^18^, Nguyen T. N. Quyen^14^, Prapat Suriyaphol^35^ & Ousman Toure^9^

**Resource Centre:** Kirk A. Rockett (Lab Projects Lead)^20^, Aaron Vanderwal (Informatics Lead)^20^, Taane Clark (Statistics Lead)^8,20^, Michael Parker(Ethics Lead)^20,25^, Rebecca Wrigley (Network Development Lead)^20^, Dominic Kwiatkowski^8,20^(Director), Daniel Alcock^8^, Sarah Auburn^8^, David Barnwell^20^, Susan Bull^20,25^, Susana Campino^8^, Jantina deVries^20,25^, Abier Elzein^17,20^, Julie Evans^20^, Kathryn Fitzpatrick^20^, Anita Ghansah^19,20^, Angie Green^20^, Lee Hart^20^, Eliza Hilton^20^, Christina Hubbart^20^, Catherine Hughes^20^, Anna E. Jeffreys^20^, Katja Kivinen^8^, Bronwyn MacInnis^8^, Magnus Manske^8^, Gareth Maslen^8^, Marilyn McCreight^20^, Alieu Mendy^20^, Catherine Moyes^20^, Claire Potter^20^, Paul Risley^7^, Kate Rowlands^20^, Miguel SanJoaquin^20,24^, Kerrin S. Small^20^, Elilan Somaskantharajah^8^, Marryat Stevens^20^, YikYing Teo^20^ & Renee Watson^20^

**Project Management Committee:** Tsiri Agbenyega^2^, Dan Carucci^36^, Katharine Cook^37^, Alan Doyle^37^, Ogobara Duombo^9^, Jeremy Farrar^14^, Michael Gottlieb^36^, Kevin Marsh^18^, Odile Puijalon^28^, Terrie Taylor^26,32^ & Dominic Kwiatkowski (Chair)^8,20^

1 The University of Buea, PO Box 63, Buea, South West Region, Cameroon.

2 Kwame Nkrumah University of Science and Technology, Private Mail Bag, Kumasi, Ghana.

3 Papua New Guinea Institute of Medical Research, PO Box 378, Madang, Papua New Guinea.

4 Swansea Medical School, Swansea University, Singleton Park, Swansea, West Glamorgan SA2 8PP, UK.

5 Institute of Child Health, College of Medicine, University of Ibadan, Ibadan, Nigeria.

6 MRC Laboratories, Atlantic Road, Fajara, PO Box 273, Banjul, Gambia.

7 National Institute for Biological Standards and Control, Blanche Lane, South Mimms, Potters Bar, Hertfordshire EN6 3QG, UK.

8 The Wellcome Trust Sanger Institute, Hinxton, Cambridge CB10 1SA, UK.

9 The Malaria Research & Training Centre, University of Bamako, PO Box 1805, Bamako, Mali.

10 London School of Hygiene & Tropical Medicine, Keppel Street, London WC1E 7HT, UK.

11 Joint Malaria Programme, Kilimanjaro Christian Medical Centre, PO Box 3010, Moshi, Tanzania.

12 Genome Science Center, Sokoine University of Agriculture, PO Box 3000, Chuo Kikuu, Morogoro, Tanzania.

13 Seattle Biomedical Research Institute, 307 Westlake Avenue North, Seattle, Washington 98109, USA.

14 Oxford University Clinical Research Unit, The Hospital for Tropical Diseases, 190 Ben Ham Tu, Quan 5, Ho Chi Minh City, Vietnam.

15 Department of Molecular Medicine, Bernhard Nocht Institute for Tropical Medicine, Postfach 30 41 2, D-20324 Hamburg, Germany.

16 Faculty of Medicine, University of Colombo, PO Box 271, Kynsey Road, Colombo 8, Sri Lanka.

17 Institute of Endemic Disease, University of Khartoum, Medical Service Science Campus, PO Box 102, Khartoum, Sudan.

18 Kenya Medical Research Institute (KEMRI)–Wellcome Trust Programme, PO Box 230, Kilifi, Kenya.

19 Noguchi Memorial Institute for Medical Research, University of Ghana, PO Box LG 581, Accra, Ghana.

20 Wellcome Trust Centre for Human Genetics, University of Oxford, Roosevelt Drive, Oxford OX3 7BN, UK.

21 National Institute for Medical Research, PO Box 9653, Dar es Salaam, Tanzania.

22 Muhimbili University of Health and Allied Sciences, PO Box 65001, Dar es Salaam, Tanzania.

23 University of Rome ‘La Sapienza’, Piazzale Aldo Moro 5, 00185 Rome, Italy.

24 Malawi–Liverpool–Wellcome Trust Clinical Research Programme, College of Medicine, University of Malawi, PO Box 30096, Chichiri, Blantyre 3, Malawi.

25 The Ethox Centre, Department of Public Health and Primary Health Care, University of Oxford, Badenoch Building, Old Road Campus, Headington, Oxford OX3 7LF, UK.

26 Blantyre Malaria Project, PO Box 32256, Chichiri, Blantyre 3, Malawi.

27 University of Maryland School of Medicine, 655 West Baltimore Street, Baltimore, Maryland 21201, USA.

28 Institut Pasteur, Unité d’Immunologie Moléculaire des Parasites, 28 Rue du Dr Roux, 75724 Paris Cedex 15, France.

29 Faculty of Tropical Medicine, Mahidol University, 420/6 Ratchawithi Road, Ratchathewi, Bangkok 10400, Thailand.

30 Centre National de Recherche et Formation sur le Paludisme, Avenue de l’Oubritenga, BP 2208, Ouagadougou 01, Burkina Faso.

31 lnstitut Pasteur de Dakar, BP 220 Dakar, Senegal.

32 Michigan State University, Department of Internal Medicine, College of Osteopathic Medicine, East Lansing, Michigan 48825, USA.

33 The Wenner-Gren Institute, Stockholm University, SE-106 91 Stockholm, Sweden.

34 Navrongo Health Research Centre, PO Box 114, Navrongo, Ghana.

35 Faculty of Medicine, Siriraj Hospital, Mahidol University, 2 Prannok road, Siriraj, Bangkoknoi, Bangkok 10700, Thailand.

36 Foundation for the National Institutes of Health, 9650 Rockville Pike, Bethesda, Maryland 20814, USA.

37 The Wellcome Trust, Gibbs Building, 215 Euston Road, London NW1 2BE, UK.

Table S1. Minor Allele Frequencies and Test of Hardy-Weinberg Equilibrium in selected candidate SNPs across ethnic groups

| **Gene** | **SNP** | **Location** | **Maj/Min** | **Bantu**  **Cases**  **(n = 385)** | **Bantu Controls**  **(n = 392)** | **Bantu**  **HWE**  **(P value)^¥^** | **Semi-Bantu**  **Cases**  **(n = 416)** | **Semi-Bantu**  **Controls**  **(n = 417)** | **Semi-Bantu**  **HWE**  **(P value)^¥^** |
| --- | --- | --- | --- | --- | --- | --- | --- | --- | --- |
| HBB | rs334 | Genic | A/T | 0.058 | 0.103 | 0.080 | 0.043 | 0.079 | 1.000 |
| IL10 | rs3024500 | P | **A/G** | 0.417 | 0.414 | 0.590 | 0.401 | 0.411 | 0.400 |
| IL10 | rs1800896 | UTR | C/T | 0.341 | 0.286 | 0.801 | 0.320 | 0.321 | 0.354 |
| IL10 | rs1800890 | UTR | A/T | 0.23 | 0.242 | 0.315 | 0.237 | 0.265 | 0.243 |
| IL1A | rs17561 | Genic | **G/T** | 0.145 | 0.152 | 0.548 | 0.117 | 0.14 | 0.030 |
| IL1B | rs1143634 | Genic | **C/T** | 0.127 | 0.117 | 0.798 | 0.125 | 0.155 | 0.173 |
| IL17RE | rs708567 | Genic | A/G | 0.49 | 0.490 | 0.051 | 0.472 | 0.499 | 0.192 |
| TLR9 | rs187084 | P | C/T | 0.245 | 0.280 | 0.507 | 0.267 | 0.31 | 0.631 |
| IL17RD | rs6780995 | Genic | A/G | 0.431 | 0.416 | 0.130 | 0.404 | 0.438 | 0.839 |
| TLR1 | rs4833095 | Genic | C/T | 0.111 | 0.097 | 0.762 | 0.108 | 0.112 | 0.603 |
| TLR6 | rs5743810 | Genic | C/T | 0 | 0.000 | NA | 0.003 | 0.001 | 1.000 |
| TLR6 | rs5743809 | Genic | C/T | 0.032 | 0.053 | 1.000 | 0.041 | 0.051 | 0.610 |
| IRF1 | rs2706384 | P | A/C | 0.348 | 0.367 | 0.418 | 0.358 | 0.402 | 1.000 |
| IL13 | rs20541 | Genic | C/T | 0.206 | 0.163 | 0.321 | 0.180 | 0.147 | 0.156 |
| IL4 | rs2243250 | I | C/T | 0.204 | 0.189 | 0.057 | 0.201 | 0.215 | 0.016 |
| LTA | rs2239704 | Genic | G/T | 0.236 | 0.243 | 0.478 | 0.250 | 0.293 | 0.617 |
| LTA | rs909253 | Genic | C/T | 0.436 | 0.439 | 0.082 | 0.426 | 0.452 | 0.414 |
| TNF | rs1799964 | P | C/T | 0.15 | 0.136 | 0.643 | 0.110 | 0.094 | 0.363 |
| TNF | rs1800750 | P | A/G | 0.07 | 0.044 | 1.000 | 0.052 | 0.038 | 0.093 |
| TNF | rs1800629 | P | A/G | 0.098 | 0.066 | 0.014 | 0.077 | 0.088 | 0.571 |
| TNF | rs361525 | P | A/G | 0.061 | 0.048 | 1.000 | 0.039 | 0.04 | 0.111 |
| TNF | rs3093662 | Genic | A/G | 0.086 | 0.087 | 0.162 | 0.075 | 0.075 | 0.151 |
| IL20RA | rs1555498 | Genic | C/T | 0.369 | 0.393 | 0.511 | 0.381 | 0.356 | 0.914 |
| TLR4 | rs4986790 | Genic | A/G | 0.074 | 0.062 | 0.636 | 0.085 | 0.058 | 0.630 |
| TLR4 | rs4986791 | Genic | C/T | 0.006 | 0.003 | 1.000 | 0.004 | 0.001 | 1.000 |
| IL22 | rs2227507 | Genic | C/T | 0.027 | 0.026 | 1.000 | 0.040 | 0.032 | 1.000 |
| IL22 | rs1012356 | Genic | A/T | 0.477 | 0.475 | 0.835 | 0.451 | 0.465 | 0.919 |
| IL22 | rs2227491 | Genic | C/T | 0.395 | 0.363 | 0.661 | 0.405 | 0.421 | 0.044 |
| IL22 | rs2227485 | P | A/G | 0.453 | 0.457 | 0.398 | 0.451 | 0.427 | 0.676 |
| IL22 | rs2227478 | P | A/G | 0.371 | 0.378 | 0.256 | 0.389 | 0.394 | 0.830 |

*UTR* 3’untranslated region, *P* promoter, Maj/ Min=Major/Minor allele**. ^¥^**One degree of freedom χ2 test of HWE applied to the controls

| **Phenotype** | **Gene** | **SNPs** | **Bantu** | | | | | | **Semi-Bantu** | | | | | |
| --- | --- | --- | --- | --- | --- | --- | --- | --- | --- | --- | --- | --- | --- | --- |
|  |  |  | **Model** | **Genotypes** | **OR** | **95% CI** | | **P value^‡^** | **Model** | **Genotypes** | **OR** | **95% CI** | | **P value‡** |
| Anaemia | hHbS | rs334 | Heterozygous | AT vs AA/TT | 0.57 | 0.26 | 1.26 | 0.162 | Heterozygous | AT vs AA/TT | 0.45 | 0.2 | 0.83 | **0.009** |
|  | IL4 | rs2243250 | Additive | TT vs CT vs CC | 0.71 | 0.45 | 1.13 | 0.150 | Additive | TT vs CT vs CC | 0.84 | 0.60 | 1.16 | 0.293 |
| CM | IL10 | rs1800896 | Additive | TT vs CT vs CC | 0.30 | 0.06 | 1.50 | 0.121 | Additive | TT vs CT vs CC | 0.41 | 0.16 | 1.06 | 0.052 |
|  | IL10 | rs3024500 | Recessive | AA vs GA/GG | 45.19 | 1.87 | 1091.2 | **0.002** | Recessive | AA vs GA/GG | 3.19 | 0.93 | 10.94 | 0.060 |
| Hyperparasite | IRF1 | rs2706384 | Heterozygous | AC vs AA/CC | 1.53 | 0.62 | 3.74 | 0.353 | Heterozygous | AC vs AA/CC | 1.97 | 0.84 | 4.63 | 0.112 |
|  | TLR1 | rs4833095 | Dominant | CT/CC vs TT | 2.23 | 0.85 | 5.83 | 0.118 | Dominant | CT/CC vs TT | 1.58 | 0.63 | 3.98 | 0.346 |
| Hyperpyrexia | IL10 | rs1800896 | Dominant | CT/CC vs TT | 0.86 | 0.43 | 1.69 | 0.658 | Dominant | CT/CC vs TT | 0.52 | 0.30 | 0.89 | 0.017 |
|  | TLR9 | rs187084 | Additive | CC vs CT vs TT | 1.62 | 0.85 | 3.06 | 0.126 | Additive | CC vs CT vs TT | 1.35 | 0.86 | 2.13 | 0.182 |
|  | IL17RD | rs6780995 | Heterozygous | GA vs GG/AA | 2.03 | 0.96 | 4.28 | 0.055 | Heterozygous | GA vs GG/AA | 1.89 | 1.09 | 3.26 | 0.021 |
| Malaria | hHbS | rs334 | Heterozygous | AT vs AA/TT | 0.47 | 0.21 | 1.09 | 0.076 | Heterozygous | AT vs AA/TT | 0.28 | 0.14 | 0.58 | **3.36 x 10^-4^** |
|  | IL17RE | rs708567 | Recessive | AA vs GA/GG | 1.50 | 0.81 | 2.79 | 0.198 | Recessive | AA vs GA/GG | 1.59 | 0.96 | 2.64 | 0.068 |
|  | IRF1 | rs2706384 | Additive | CC vs AC vs AA | 0.81 | 0.55 | 1.19 | 0.285 | Additive | CC vs AC vs AA | 0.73 | 0.53 | 1.01 | 0.054 |
|  | TLR9 | rs187084 | Additive | CC vs CT vs TT | 0.96 | 0.63 | 1.48 | 0.869 | Additive | CC vs CT vs TT | 1.00 | 0.74 | 1.39 | 0.983 |
|  | IL13 | rs20541 | Dominant | CT/CC vs TT | 0.81 | 0.47 | 1.43 | 0.472 | Dominant | CT/CC vs TT | 1.34 | 0.84 | 2.14 | 0.225 |
| SMA | hHbS | rs334 | Recessive | TT vs AT/AA | 298.33 | 1.86 x 10^-32^ | 4.79 x 10^36^ | 0.676 | Recessive | TT vs AT/AA | 234.03 | 26.63 | 2056.4 | **1.41 x 10^-6^** |
| UM | IL17RE | rs708567 | Heterozygous | GA vs GG/AA | 0.74 | 0.40 | 1.38 | 0.344 | Heterozygous | GA vs GG/AA | 0.42 | 0.22 | 0.79 | **0.007** |
|  | IL17RD | rs6780995 | Dominant | GA/AA vs GG | 1.17 | 0.48 | 2.86 | 0.734 | Dominant | GA/AA vs GG | 3.02 | 1.06 | 8.56 | 0.023 |

Table Table S2. Results of genotype associations between selected SNP and syndromes of malaria in the two major ethnic groups

**^‡^**We performed additive, dominant, recessive and heterozygous advantage genotypic tests, adjusted for age, sex, ethnicity and the HbS polymorphism
